# Downregulated INHBB in endometrial tissue of recurrent implantation failure patients impeded decidualization through the ADCY1/cAMP signalling pathway

**DOI:** 10.1007/s10815-023-02762-7

**Published:** 2023-03-13

**Authors:** Hui Zhang, Zhilong Wang, Quan Zhou, Zhiwen Cao, Yue Jiang, Manlin Xu, Jingyu Liu, Jidong Zhou, Guijun Yan, Haixiang Sun

**Affiliations:** 1 Department of Obstetrics and Gynecology, Center for Reproductive Medicine, Affiliated Drum Tower Hospital, Medical School of Nanjing University, Nanjing, China; 2 State Key Laboratory of Pharmaceutical Biotechnology, Nanjing University, Nanjing, China; 3 Center for Molecular Reproductive Medicine, Nanjing University, Nanjing, China; 4 State Key Laboratory of Reproductive Medicine, Nanjing Medical University, Nanjing, China

**Keywords:** INHBB, Decidualization, RIF, cAMP, ADCY1

## Abstract

**Purpose:**

This study aims to identify the mechanism of Inhibin Subunit Beta B (INHBB), a member of the transforming growth factor-β (TGF-β) family involved in the regulation of human endometrial stromal cells (HESCs) decidualization in recurrent implantation failure (RIF).

**Methods:**

RNA-seq was conducted to identify the differentially expressed genes in the endometria from control and RIF patients. RT-qPCR, WB, and immunohistochemistry were performed to analyse the expression levels of INHBB in endometrium and decidualised HESCs. RT-qPCR and immunofluorescence were used to detect changes in the decidual marker genes and cytoskeleton after knockdown INHBB. Then, RNA-seq was used to dig out the mechanism of INHBB regulating decidualization. The cAMP analogue (forskolin) and si-INHBB were used to investigate the involvement of INHBB in the cAMP signalling pathway. The correlation of INHBB and ADCY expression was analysed by Pearson’s correlation analysis.

**Results:**

Our results showed significantly reduced expression of INHBB in endometrial stromal cells of women with RIF. In addition, INHBB was increased in the endometrium of the secretory phase and significantly induced in in-vitro decidualization of HESCs. Notably, with RNA-seq and siRNA-mediated knockdown approaches, we demonstrated that the INHBB-ADCY1-mediated cAMP signalling pathway regulates the reduction of decidualization. We found a positive association between the expression of INHBB and ADCY1 in endometria with RIF (*R*^2^ = 0.3785, *P* = 0.0005).

**Conclusions:**

The decline of INHBB in HESCs suppressed ADCY1-induced cAMP production and cAMP-mediated signalling, which attenuated decidualization in RIF patients, indicating that INHBB is an essential component in the decidualization process.

**Supplementary Information:**

The online version contains supplementary material available at 10.1007/s10815-023-02762-7.

## Introduction

Despite technical advances in assisted reproductive technology (ART), the management of recurrent implantation failure (RIF) poses an unmet clinical challenge that affects approximately 10% of women who have undergone several in vitro fertilization-embryo transfers (IVF-ETs) and still has an extremely low success rate [1, 2]. RIF refers to the condition in which good-quality embryos repeatedly fail to implant after two or more IVF cycles with unclear aetiology [3, 4]. Current studies tend to assume that RIF occurs due to defects in early pregnancy events, including implantation and decidualization [5].

Decidualization, known as the progesterone-dependent differentiation of fibroblast-like human endometrial stromal cells (HESCs) into large, secreting decidual cells, is a key step to achieve successful implantation. This process occurs in the middle to late phase of the menstrual cycle, and subsequent embryo implantation leads to and extends the persistent decidualization throughout the endometrium, forming the pregnancy decidua to support the development of the embryo [6]. Decidualised cells are highly secretory and secrete prolactin (PRL) and insulin-like growth factor-binding protein 1 (IGFBP1) as decidualization markers [7, 8]. It is believed that multiple signalling and regulatory pathways are involved in decidualization, such as the progesterone, prostaglandin E2, and cyclic adenosine monophosphate (cAMP)/protein kinase A (PKA) signalling pathways [7, 9]. In culture, HESCs display a decidual phenotype when treated with progestins, which could be enhanced by cAMP analogues [10, 11]. Adenylate cyclases (ACs), membrane-bound proteins that convert adenosine triphosphate into cAMP, control the production of cAMP [12], which could subsequently activate PKA and cAMP-responsive element-binding protein to induce the morphological and biochemical characteristics of decidualization [13].

The βB-subunit of the inhibin gene (INHBB) encodes a preprotein subunit of inhibin/activin, which are functional cytokines belonging to the transforming growth factor-β (TGF-β) family [14–16]. A previous study demonstrated that INHBB mRNAs were expressed in human decidualised endometrium from the first trimester of pregnancy and increased as the gestation progressed [17]. Furthermore, McConaha et al. [18] indicated that INHBB expression increases in the mouse uterus in areas undergoing decidualization. These studies indicated that INHBB might play essential roles in endometrial decidualization. However, the functions of INHBB on decidualization are still unknown.

In the present study, we identified that INHBB was downregulated in secretory-phase endometrium from RIF patients. The aim of this study was to clarify the possible roles of INHBB in the pathophysiology of RIF and define the underlying molecular mechanisms of INHBB in decidualization. We demonstrated that decreased INHBB inhibited ADCY1 expression and impaired cAMP signalling, thus attenuating decidualization in RIF patients.

## Materials and methods

### Study participants

The Medical Ethics Committee of Nanjing Drum Tower Hospital approved this study (No. 2013–081-01). Written informed consent was obtained from each participant. Between January 2020 and November 2021, 28 infertile women aged 20–40 years with normal and regular menstrual cycles (25–35 days) and no history of steroid hormone medication in the last 3 months were included in the study. The exclusion criteria included the following: patients with (a) PCOS, (b) hydrosalpinx, (c) endometrial polyps, (d) untreated moderate to severe intrauterine adhesions, endometriosis, and submucosal uterine fibroids, (e) abnormal thyroid or immune function, and (g) chromosomal abnormalities. The control group consisted of healthy fertile women who seek for ART due to male factors and achieved clinical pregnancy after the first or second IVF cycle. The RIF group included patients with at least three failures of high-quality blastocyst transfer cycles at the reproductive medicine centre of Nanjing Drum Tower Hospital. The secretory endometrium was obtained at 5–7 days after ovulation monitored by ultrasound. The proliferative endometrium was obtained from 6 premenopausal, nonpregnant patients undergoing hysterectomy for benign diseases. Decidual tissue was obtained from induced abortions with no medical indication. There was no significant difference in average age or body weight index between the two groups. Patient information statistics are shown in the Supplementary Table 1.

### RNA-sequencing analysis

Transcriptome sequencing was performed as described previously [5, 19], and brief descriptions are as follows. Endometrial tissue samples of the mid-secretory phase were obtained via pipe suction curettage 5–7 days after ovulation from the control group (*n* = 3) and RIF group (*n* = 3) (SRP224538). HESCs were cultured in 6 cm dishes, and when the cells reached a confluence of 90%, decidualization was induced for 3 days (SRP224679). HESCs were cultured in 6 cm dishes and transfected with the indicated si-INHBB for 2 days, then decidualised for another 3 days. The total RNA of these cells was extracted with TRIzol reagent (Invitrogen), and the cDNA libraries were constructed from 1 μg of total RNA and then sequenced by OE Biotech Co., Ltd. (Shanghai, China). High-quality reads were mapped to reference genome (GRCh38.p12) using hisat2, and the DESeq2 (v1.29.12) package was used to normalise count data and for differential gene expression (log2FC > 1, *P* value < 0.05) analysis. KEGG, GO, and GSEA was performed using the clusterProfiler package [20]. The TCseq package was used to analyse the dynamic gene expression in different groups with the R program (R version 4.0.2).

### Immunohistochemistry (IHC)

Animal experiments were performed under the supervision of the Laboratory Animal Management Committee (Jiangsu Province, China). The Nanjing Drum Tower Hospital Institutional Animal Care and Use Committee approved this application (SYXK 2019–0058) and the ICR mice were maintained in Nanjing Drum Tower Hospital's Experimental Animal Center on a 12/12 h light/dark cycle (lights off at 19:00). The morning of a vaginal plug was observed, mice were deemed to be at gestational stage dpc0.5. Paraffin-embedded tissue blocks were sectioned into 4-μm-thick sections, which were then subjected to IHC as described previously, with some modifications [21]. Tissue sections were deparaffinised, rehydrated, and exposed to 3% v/v H_2_O_2_, then incubated with primary rabbit-anti-INHBB antibody (Proteintech) at 4 °C overnight. Immunostaining was performed using immunohistochemical staining kits (Zhongshan Golden Bridge) according to the manufacturer’s instructions. The slides were captured by Leica DM 2000 microscope and LAS Core software (Leica Microsystems Limited) with the magnification 200 × . The INHBB staining intensities were evaluated by the mean of integrated optical density (IOD) which was calculated from IOD/Area.

### Cell culture and materials

HESCs from ATCC were seeded at a density of 5 × 10^5^ cells/well in 6-well plates within Dulbecco’s Modified Eagle Medium/Nutrient Mixture F-12 (DMEM-F12) medium supplemented with 10% foetal bovine serum (FBS) and 1% penicillin–streptomycin. HESCs were transfected with Lipofectamine 3000 (Invitrogen) and siRNA pool targeting INHBB (si-INHBB) or a non-targeting control (si-CTL) at a final concentration of 50 nM for 2 days and then treated with 0.5 mM 8-Br-cAMP (Sigma) and 1 μM MPA (Sigma) in DMEM-F12 (Gibco) with 2.5% charcoal/dextran-treated FBS (HyClone) to induce decidual differentiation. Every 24 h, HESCs were observed under an inverted microscope to study their morphological features. Specifically, HESCs were stimulated with 1 mM 8-Br-cAMP or 2.5 μM forskolin (cAMP activator) for the functional rescue of INHBB knockdown. The culture medium was collected at 72 h and 144 h, and the supernatants were collected to detect prolactin (PRL) as describe previously [5].

### Immunofluorescence staining for F-actin filaments

HESCs grown in 24-well plates were treated with si-INHBB for 2 days, and then exposed to a decidualization stimulus of 8-Br-cAMP plus MPA for 2 days; they were fixed with 4% paraformaldehyde (w/v) for 30 min at room temperature. Cells were then washed with PBS and permeabilised at room temperature with 0.5% Triton X-100 in PBS. Subsequently, the cells were blocked with 3% BSA in PBS and incubated with Amanita phalloides (1:200; P5282, Sigma) at 4 °C overnight. Fluorescence confocal microscopy (Olympus, FV10i) was used to capture images after the nuclei had been stained with 4′,6-diamidino-2-phenylindole dihydrochloride (DAPI) (Sigma).

### cAMP assay

Total cAMP levels in HESCs were determined using a competitive ELISA kit (#ab65355, Abcam) according to the manufacturer’s recommendations. Cells were lysed for 20 min in 282 μl of 0.1 M HCl and were centrifuged at 13,500 rpm at 4 °C. Then, 20 μl of supernatant was used for the measurement. The sample was diluted and mixed with acetylating reagents. After that, the samples were loaded in 96-well plates and incubated with cAMP antibody for an hour. cAMP-HRP was added to each well, incubated for another hour and washed 3 times with wash buffer. Subsequently, HRP developer was added and incubated for 1 h. The reaction was stopped by 1 M HCl, and the colour developed was read at OD 450 nm using a plate reader (Scientific MuLtiskan SpectruM, Thermo).

### Western blot analysis

Endometrial tissue and HESCs were lysed in RIPA buffer with a protease inhibitor cocktail (Sigma) and a phosphatase inhibitor cocktail (Sigma), then quantified with BCA protein assay. Twenty μg of proteins from cell lysate or tissue homogenate were separated by 12% SDS-PAGE, then transferred to a 0.22 μm PVDF membrane (Millipore) by a wet protein transfer system for 90 min in ice baths. After blocking the membrane for 1 h at room temperature with 5% skimmed milk (BIO-RAD) in TBS-0.05%, specific antibodies against INHBB (Proteintech), ACTB (Bioworld) were detected using the appropriate secondary antibodies and visualised using a ChemiScope Mini CLINX (Shanghai Kerui Biotechnology). For quantification, western blot bands were normalised to the intensity of corresponding ACTB band densities using ImageJ software (version 1.61).

### Quantitative reverse transcription real-time PCR (RT-qPCR)

TRIzol reagent was used to extract total RNA from endometrial biopsies or HESCs. A total of 1 μg of extracted RNA was transcribed into cDNA using 5 × All-In-One RT Master Mix (Abm). cDNA was mixed with primers and SYBR Green Master (Vazyme) and measured by MyiQ real-time PCR studios (BIO-RAD). The primers were designed using Primer-BLAST, and the primer sequences are provided in Supplementary Table 2 at 10 μM working stocks.

### Statistical analysis

All the data represent assays performed in three or more replicates. The data normally distributed are expressed as the means ± SEM. GraphPad Prism 6.0 software was used to analyse data. Statistical comparisons between the 2 groups were performed using Student’s test. Analysis of variance (ANOVA) was used for statistical comparisons among 3 or more groups, followed by Tukey’s multiple comparisons. The correlation analyses were performed using Pearson’s correlation. *P* value < 0.05 was considered statistically significant without extra notation.

## Results

### Transcriptional profile of RIF patients associated with dysregulation of decidualization

**Fig. 1:**
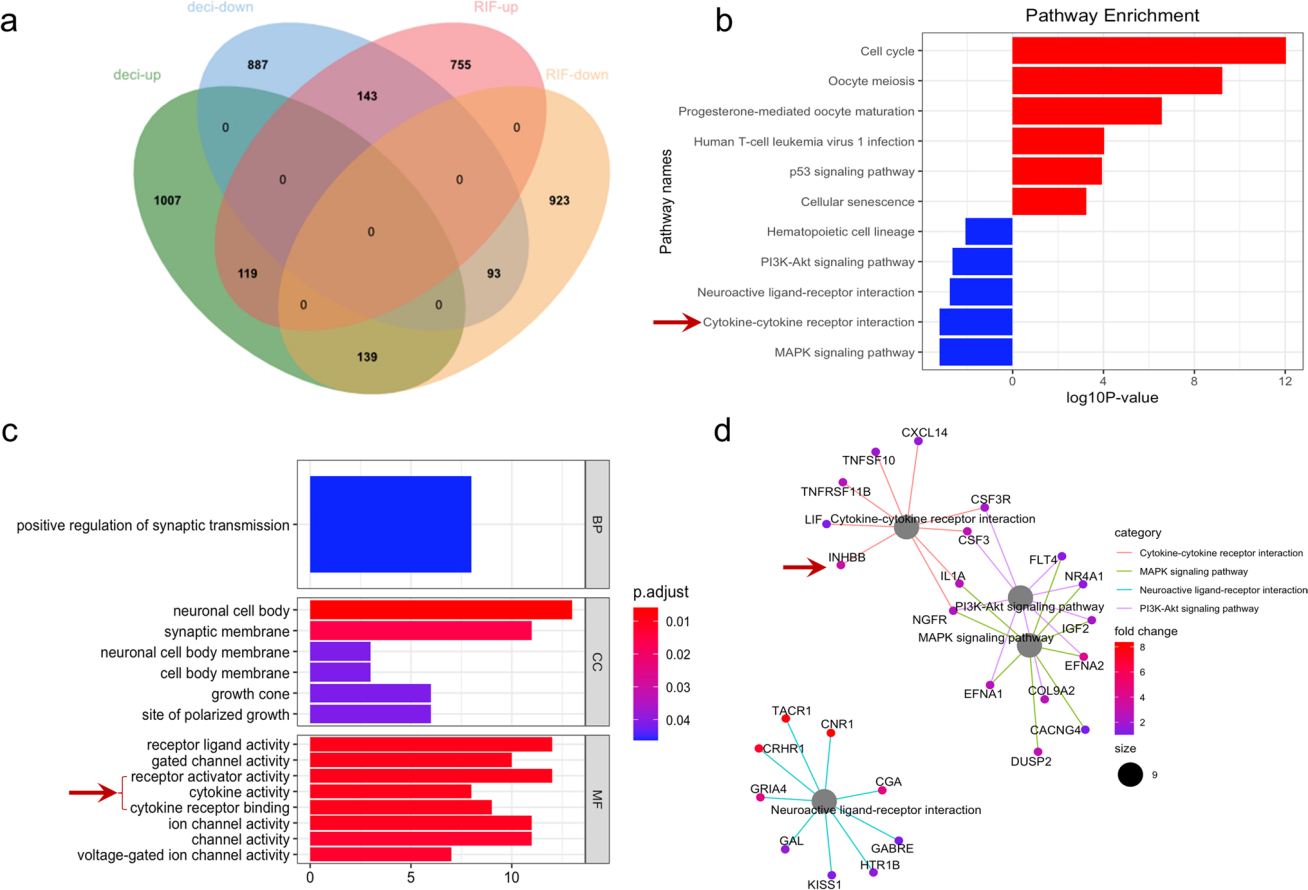
Transcriptome sequencing and bioinformatics analysis for patients with recurrent implantation failure (RIF). **a** Venn diagram indicating the intersected target genes identified by RNA-seq analysis of decidualization process (SRP224679) and RIF endometria (SRP224538). Log2FC > 1 and *P* value < 0.05 values were considered to determine the differentially expressed genes (DEGs). **b** and **c** KEGG pathway and Gene Ontology (GO) enrichment analysis of DEGs. **d** The DEGs related to the altered KEGG pathways

To gain insight into the role of the endometrium in the pathogenesis of RIF, we analysed our RNA-seq data of RIF endometria (SRP224538) combined with the transcriptome of HESCs decidualization (SRP224679). The Venn diagram showed that 139 genes were highly expressed in the decidualization process while downregulated in RIF patients; whereas another 143 genes decreased during decidualization were upregulated in RIF (Fig. 1a). KEGG pathway and GO enrichment analysis were performed with these 282 genes (Fig. 1 b–d). The top enriched KEGG pathway terms included ʻcell cycleʼ, ʻcytokine-cytokine receptor interactionʼ, and ʻMAPK signalling pathwayʼ. The results of the Gene Ontology (GO) analysis enriched in cytokine activity and cytokine receptor binding. Consistent with published studies, multiple genes (such as CNR1, NR4A1 and IL1A) were involved in our results [22, 23]. We also found that INHBB was differentially expressed in decidualised stromal cells and the RIF endometria (Fig. 1d); however, the mechanism has not been elucidated.

### Downregulated INHBB in RIF patients

**Fig. 2:**
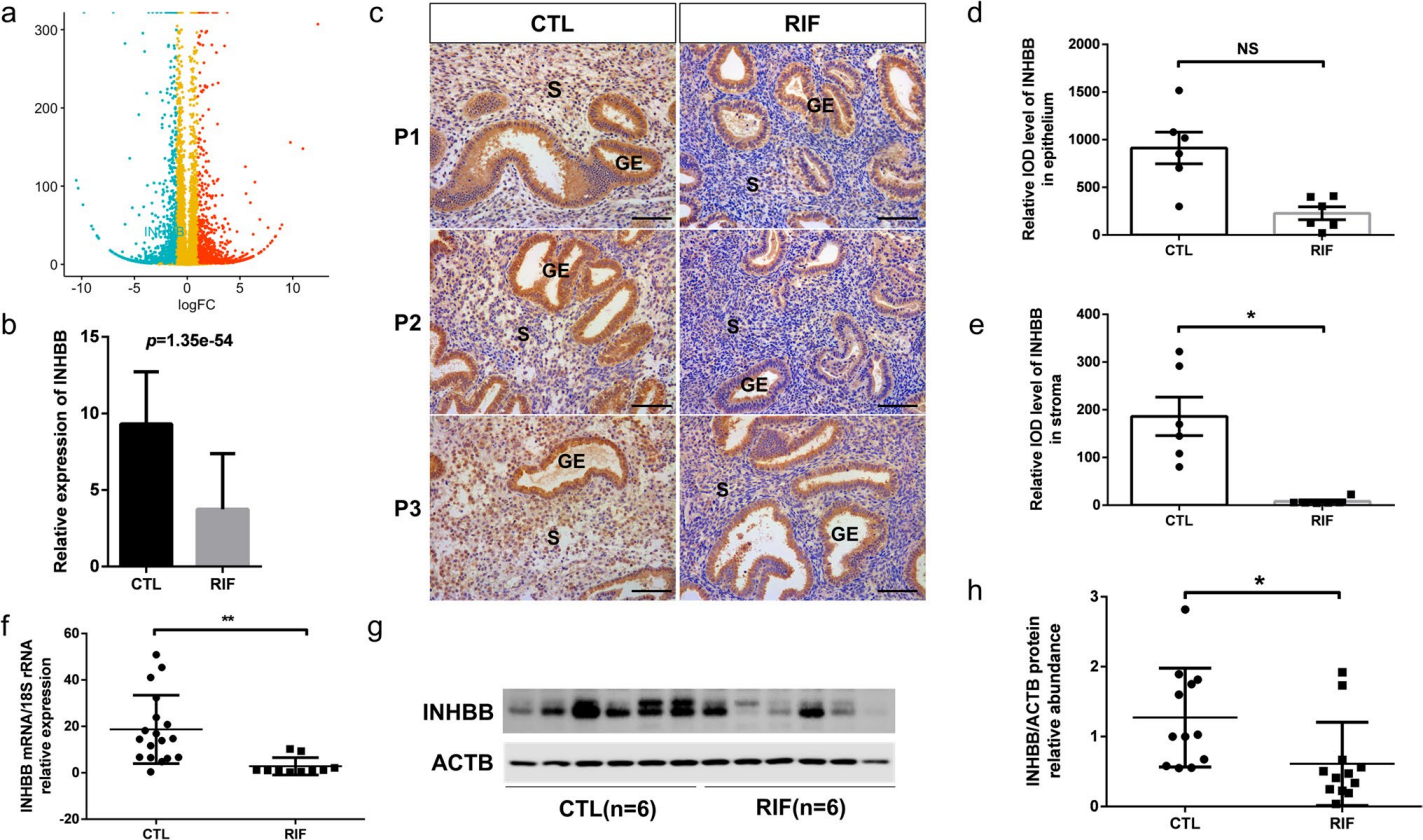
Downregulated INHBB in RIF patients. **a** Volcano plots of DEGs compared with RIF and control women (change > two fold, adjusted* P* value < 0.05) (*n* = 3 vs 3). **b** Transcriptome levels (FPKM) of INHBB. **c** IHC staining of INHBB in the endometria of control fertile (CTL) and RIF patients. P1, P2, and P3 indicate three different women. Scale bar = 100 μm. S, stroma; GE, glandular epithelium. **d** The mean IOD of INHBB of the epithelial part. IOD, integrated optical values, analysed by Image-Pro Plus 6. NS: no statistical difference. **e** The mean IOD of INHBB of the stromal part. *P < 0.05. **f** The relative mRNA expression of INHBB was quantified using RT-qPCR. Expression levels are shown relative to 18S rRNA; CTL (*n* = 18) and RIF (*n* = 10). **P < 0.01. **g** The protein levels of INHBB in CTL and RIF were assessed using western blotting. **h** The relative protein levels of INHBB were quantified. CTL (*n* = 12) and RIF (*n* = 12). *P < 0.05

Through analysing the RNA-seq data, we found that INHBB expression level was downregulated in the endometria of RIF patients (Fig. 2a and b). Further, the expression and location of INHBB in the endometrium were detected by IHC (Fig. 2c). The mean INHBB integrated optical density (IOD), especially in stromal cells relative to epithelial cells (Fig. 2d and e), was significantly lower in the RIF group (*P* = 0.0381). RT-qPCR results also revealed decreased expression of INHBB in RIF patients (CON vs RIF: 18.675 ± 3.473, *n* = 18 vs 2.812 ± 1.183, *n* = 10) (Fig. 2f). The relative levels of INHA and INHBA mRNA in RIF patients were shown in supplementary Fig. 1. We also found that INHA was decreased in the RIF endometrium (CON vs RIF: 2.110 ± 0.2797, *n* = 14 vs 0.8933 ± 0.1978, *n* = 11), while the magnitude of discrepancy was not significant as INHBB. In addition, we examined the protein expression of INHBB and found a lower expression in RIF patients (Fig. 2g and h).

### Spatiotemporal expression of INHBB in the endometrium

**Fig. 3:**
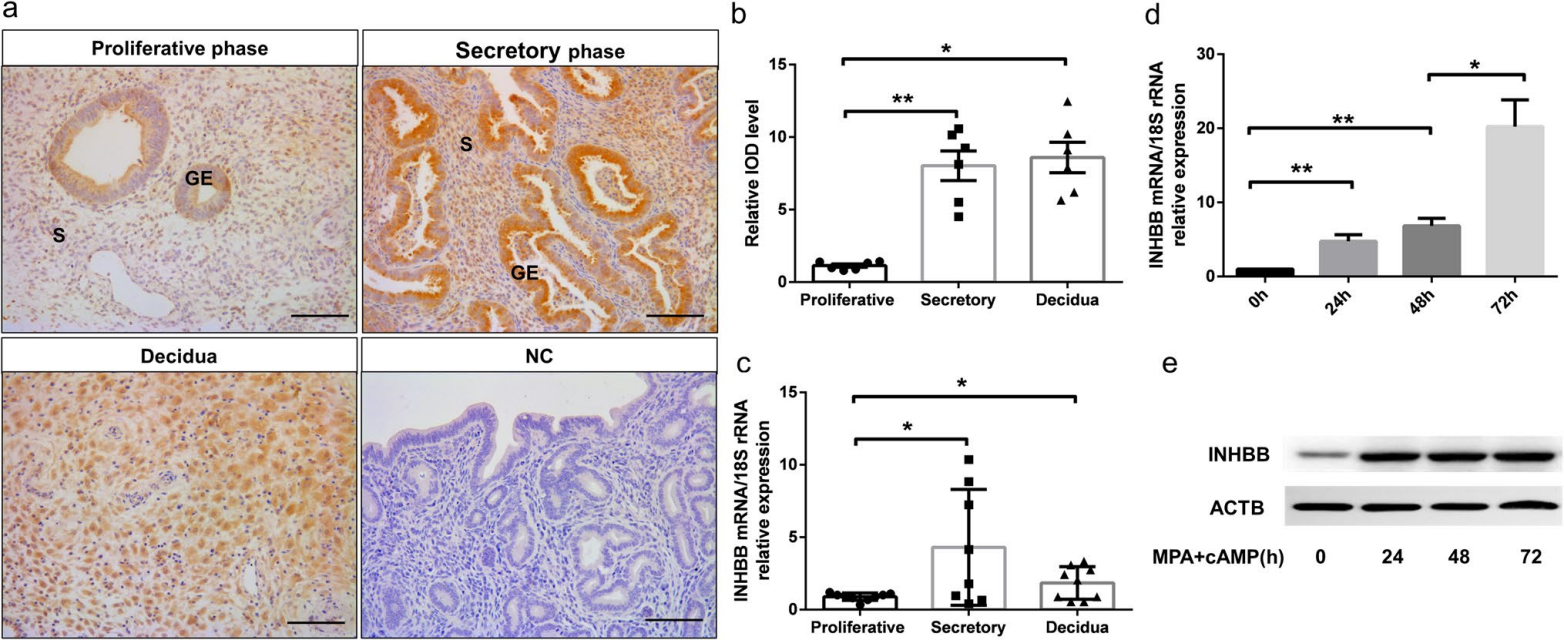
Spatiotemporal expression of INHBB in the endometrium and HESCs. **a** Expression of INHBB in the endometrium. Scale bar = 100 μm. S, stroma; GE, glandular epithelium. **b** Quantifying IHC results from the stromal compartment of the endometrium (*n* = 6 for each group). **P < 0.05, *P < 0.05, using one-way ANOVA. **c** RT-qPCR was performed to assay the expression of INHBB during physiological cycles (*n* = 9 for the Proliferative phase and Decidua groups, and *n* = 8 for the Secretory phase group). *P < 0.05, using one-way ANOVA. **d** The expression pattern of INHBB in HESCs treated with 0.5 mM 8-Br-cAMP and 1 μM MPA for different periods (0, 24, 48, or 72 h) was evaluated by RT-qPCR, *P < 0.05, **P < 0.01, ***P < 0.001 (*n* = 3, one-way ANOVA). **e** Western blot analysis was used to examine the protein levels of INHBB in HESCs treated with 0.5 mM 8-Br-cAMP and 1 μM MPA for different periods (0, 24, 48, or 72 h)

To demonstrate a putative function of INHBB in physiological status, we detected INHBB expression during the normal menstrual cycle. We found that INHBB was expressed in the cytoplasm and rose in the secretory phase and first-trimester decidua (Fig. 3a). Further analysis showed the mean IOD of INHBB was lower (*P* < 0.05) during the proliferative phase than a secretory and decidual phase. No significant difference in the staining intensity was observed between the latter two (Fig. 3b). INHBB mRNA expression levels exhibited a consistent trend with the IHC (Fig. 3c). Furthermore, INHBB exhibited increased numbers of positive cells at the peri-implantation stage (E0.5–7.5), accompanied by progressed decidualization in ICR mice (Supplementary Fig. 2). These results suggested a potential role for INHBB during endometrial decidualization. To analyse the expression of INHBB during decidualization in vitro, we used cAMP and MPA to induce decidualization of HESCs. The protein amount increased with prolonged induction, suggesting that INHBB plays an important role in human decidualization (Fig. 3d and e).

### Silencing of INHBB reduced the induction of decidualization

**Fig. 4:**
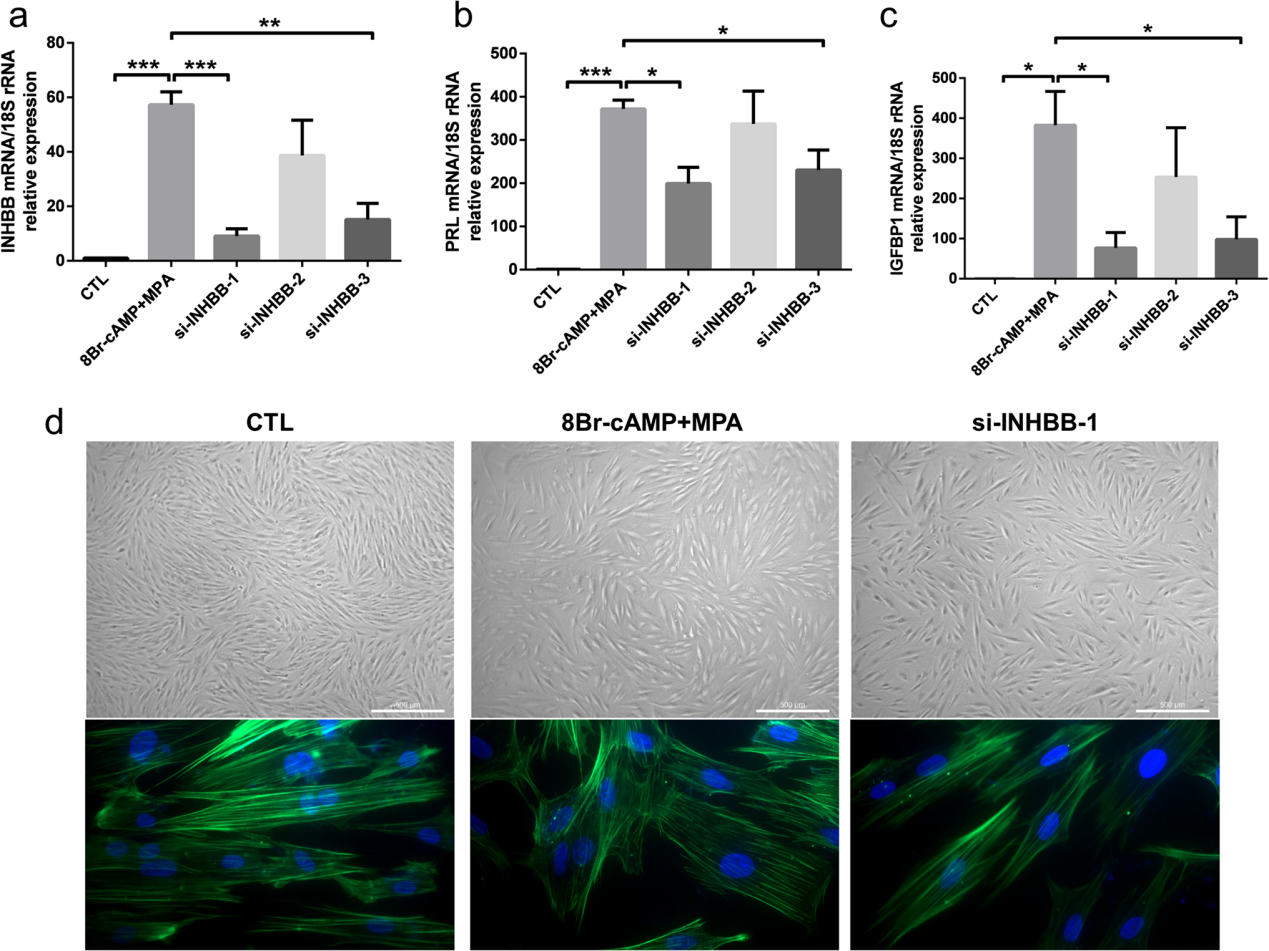
Knockdown of INHBB inhibited decidualization of HESCs. **a**–**c** HESCs were transfected with si-INHBB or si-CTL for 48 h and then treated with 0.5 mM 8-Br-cAMP and 1 μM MPA for decidualization stimulus. The expression of INHBB (**a**), PRL (**b**) and IGFBP1 (**c**) were measured by qPCR, respectively. *P < 0.05, **P < 0.01, ***P < 0.001 (*n* = 3, one-way ANOVA). si-INHBB-1, -2, and -3, different target sites of the si-RNA. **d** Immunofluorescence analyses of F-actin were visualised using the green-fluorescent Alexa Fluor® 488 phalloidin at 48 h of in vitro decidualization after treatment with vehicle or si-INHBB-1 in HESCs. The upper panel (magnification 10 × , scale bar = 500 μm) is the bright field; the lower panel is the fluorescent and merged view of HESCs (magnification 400 ×). The experiment was performed in triplicates independently

To demonstrate the reverse role of INHBB, we knocked down INHBB with siRNA in HESCs. Three siRNA sequences were designed, and the optimal knockdown effect was observed in the si-INHBB-1 sequence with a maximum knockdown efficiency of 90% after transfecting the targeted siRNA for two days (Fig. 4a). We then found that INHBB knockdown impaired HESC decidualization, as evidenced by decreased PRL and IGFBP1 expression (Fig. 4b, c). Decidualization is accompanied by morphological changes from an elongated spindle phenotype to a polygonal and randomly arranged shape. In contrast, the knockdown of INHBB displayed a noticeable appearance with parallel intracellular actin fibres, which differs from the expanded web-shaped actin fibres of decidualised HESCs (Fig. 4d). Considering all these results, we confirmed that INHBB is important during decidualization, and its deficiency led to defective decidualization.

### Transcriptome sequencing of HESCs with INHBB knockdown

**Fig. 5:**
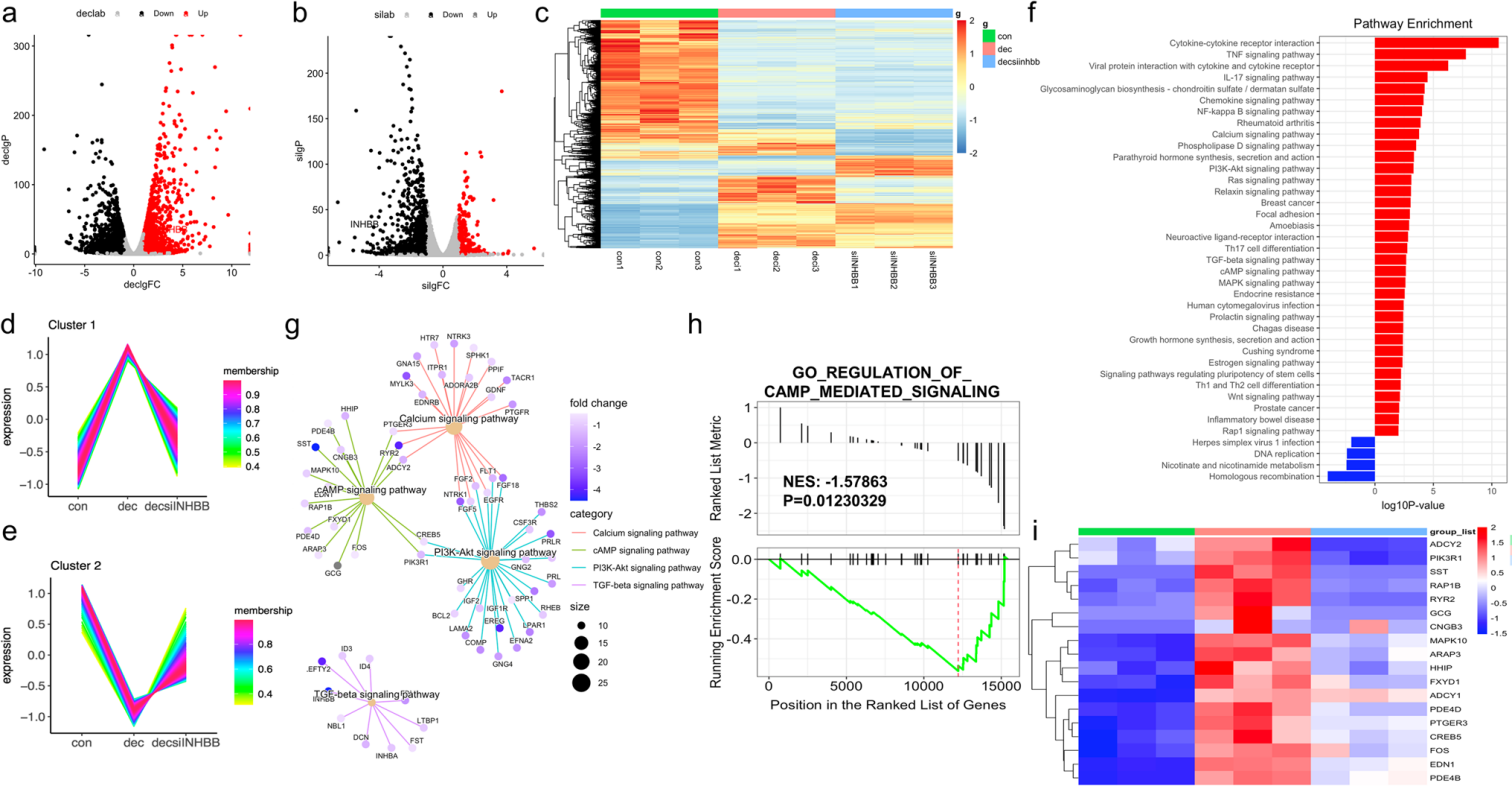
Transcriptome sequencing of INHBB silencing. **a** Volcano plot showing DEGs (fold change > 2; *p* value < 0.05) between the control and decidual tissue following in vitro decidualization. **b** Volcano plot of decidual DEGs (fold change > 2; *P* value < 0.05) after INHBB knocked down. **c** Heatmap of the clustering expression profiles of DEGs in the three groups. con, control group; dec, decidualization group; decsiinhbb, decidualization with INHBB knockdown group. **d** Clustering of genes that upregulated in decidualization while downregulated by INHBB silencing. **e** Clustering of genes that induced in decidualization while decreased by INHBB silencing. **f**, **g** KEGG enrichment analysis of the genes from cluster1 (*n* = 454) and cluster2 (*n* = 627). **h** GSEA plot. The analysis was performed against the KEGG database of the cAMP signalling pathway. The *x*-axis represents the rank for all genes; the *y*-axis represents the value of the ranking metric. **i** Frequency heatmap of the DEGs belonging to the cAMP signalling pathway

Then, RNA-seq was performed to analyse the possible mechanism of INHBB in the regulation of decidualization. The different expressed genes (DEGs) were presented with a volcano plot and heatmap (Fig. 5 a–c), and INHBB was noted in the graph. TCseq package was used to separate the gene profile into 6 clusters according to their expression tendencies (Fig. 5d, e and Supplementary Fig. 3). The Cluster 1 (*n* = 454) and Cluster 2 (*n* = 627) were selected for the following KEGG analysis. Pathway enrichment analysis enriched many pathways such as the calcium signalling pathway, the cAMP signalling pathway, the PI3K-AKT signalling pathway, and the TGF-β signalling pathway (Fig. 5f and g), of which the cAMP signalling pathway is important for the decidualization [24]. Notably, GSEA highlighted that cAMP-mediated signalling was significantly downregulated when INHBB was knocked down during decidualization (Fig. 5h). In this pathway, 18 genes were significantly enriched and are further displayed according to a heatmap (Fig. 5i). Among these genes, we found that adenylate cyclase ADCY changed significantly (Fig. 5i and Supplementary Fig. 4).

### Suppression of INHBB reduced cAMP signalling and impaired decidualization

**Fig. 6:**
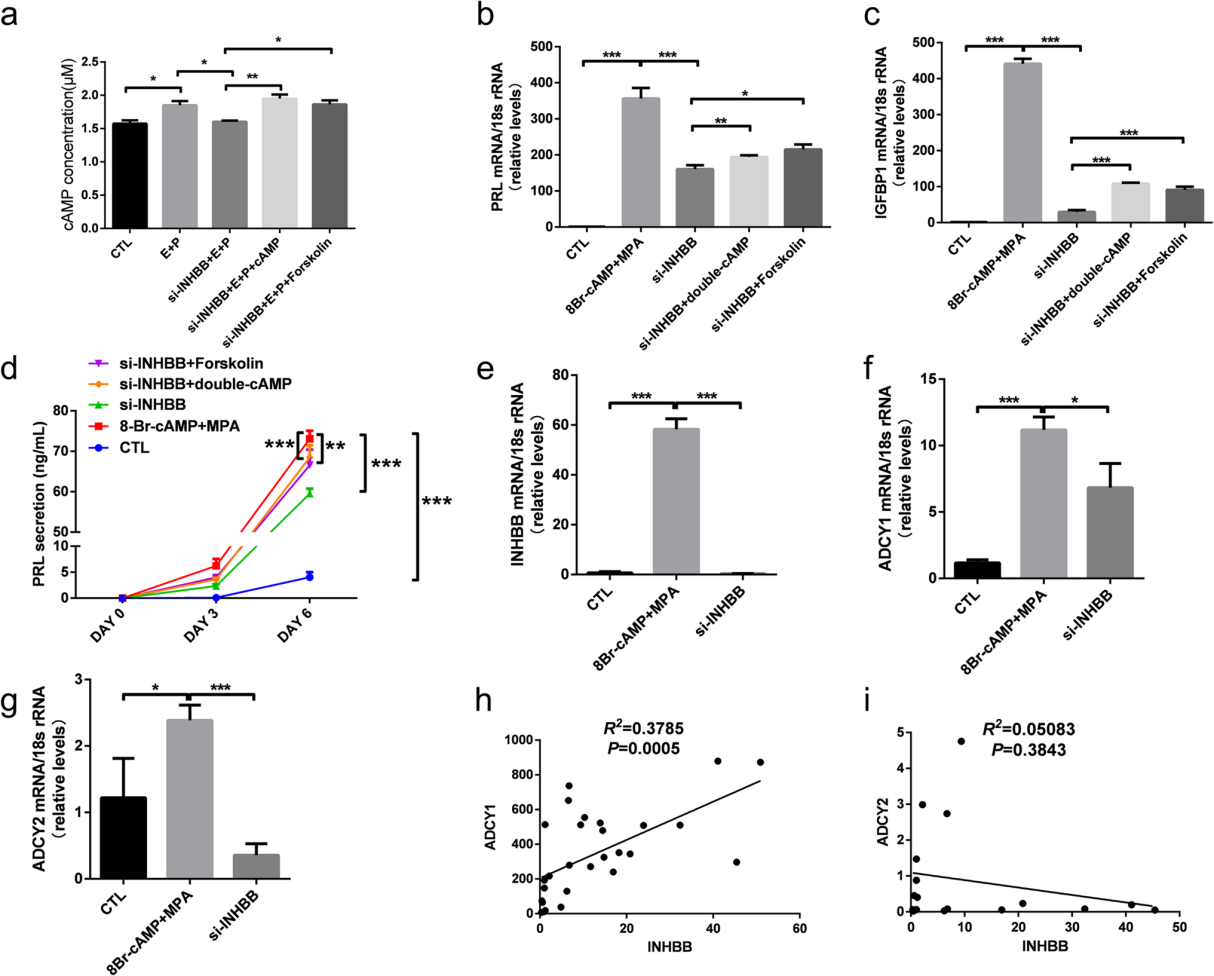
Reduction of INHBB inhibited ADCY1 and impaired decidualization. **a** HESCs were transfected with si-INHBB or si-CTL for 48 h and then treated with 38 nM E2 (E) and 1 μM MPA (P) for inducing differentiation. HESCs were stimulated with 1 mM 8-Br-cAMP or 2.5 μM forskolin (cAMP activator) for 12 h before we harvested the differentiated HESCs. Intracellular cAMP concentrations determined by cAMP-specific ELISA kit. The experiments were performed three times independently, *p < 0.05, **p < 0.01, using one-way ANOVA. The mRNA levels of PRL (**b**) and IGFBP1 (**c**) from these groups were examined using RT-qPCR. *p < 0.05, **P < 0.01, *** *P* < 0.001 (*n* = 3, one-way ANOVA). **d** Prolactin secretion was measured by ELIFA. * *P* < 0.05, ** *P* < 0.01, *** *P* < 0.001 (*n* = 3, two-way ANOVA). **e**–**g** The mRNA levels of INHBB (**e**), ADCY1 (**f**) and ADCY2 (**g**) were examined using RT-qPCR. *P < 0.05, ***P < 0.001 (*n* = 3, one-way ANOVA). **h** Pearson correlation analysis of mid-secretory endometrial protein levels of INHBB and ADCY1 mRNA levels (*R*^2^ = 0.3785, *P* = 0.0005) in all samples (*n* = 28). **i** Pearson correlation analysis of mid-secretory endometrial protein levels of INHBB and ADCY2 mRNA levels (*R*^2^ = 0.0508, *P* = 0.3843) in all samples (*n* = 17)

As ADCY promotes cAMP production and activation of cAMP signalling, we investigated whether the downstream effector of INHBB would influence intracellular cAMP contents. We found that INHBB knockdown reduced the level of cAMP secreted by HESCs (Fig. 6a). Interestingly, these could be rescued by direct supplementation with the cAMP or cAMP agonist forskolin (Fig. 6a), and the same with the secretion of PRL and IGFBP1, the marker genes of decidualization (Fig. 6 b–d). Besides, the reduction of ADCY by INHBB silencing was verified by RT-PCR (Fig. 6 e–g). Then the correlation coefficients between ADCY and INHBB indicated that ADCY1 may be the target gene regulated by INHBB (Fig. 6h and i), which is consistent with others reported sequencing results (Supplementary Fig. 5). The results suggested that suppression of the INHBB level reduced ADCY1, resulting in a decreased level of cAMP and leading to attenuated decidualization (Supplementary Fig. 6).

## Discussion

In the present study, we revealed that INHBB was reduced in the endometria of RIF patients and participate in the regulating of decidualization, and downregulation of INHBB impaired cAMP signalling pathway mediated by ADCY1. In addition, this defective effect could be counteracted in vitro by the elevation of cAMP levels or by specific activation of cAMP by forskolin.

Our preliminary transcriptional profiling analyses showed that cytokine and cytokine receptor signalling pathways have a role in physiological and pathophysiological changes in secretory endometria of women with RIF, which is consistent with previously reported findings that cytokine and cytokine receptor signalling participate in endometrial decidualization [5, 25, 26]. TGF-β superfamily members, multifunctional cytokines, are abundantly expressed in the endometrium and regulate multifaceted reproductive processes [27–29]. Activins and inhibins have been found to play roles in the paracrine regulation of endometrial receptivity, decidualization, and implantation [30, 31]. The production of activin A is induced in HESCs in response to cAMP signalling and increases in parallel with PRL secretion. It is generally accepted that activin A, a positive regulator of decidualization, promotes the expression of PRL and IGFBP1 and enhances the secretion of interleukin-8 and vascular endothelial growth factor from endometrial cells to regulate endometrial angiogenesis [32–34]. In addition, it was shown that activin A and inhibin A differentially regulate the expression of MMPs and activin A signalling involved in the remodelling associated with implantation and foetomaternal interaction, which correlates directly with the thickness of the endometria [32]. To further examine the pathophysiological significance of these growth factors in RIF patients, we investigated the variations in the subunits and explored the regulatory mechanism involved.

Activin is a homodimer composed of two β subunits (activin A: βA/βA, activin AB: βA/βB, activin B: βB/βB). Inhibin is a dimer of the α and β subunit proteins (inhibin A: αβA, inhibin B: αβB). Low expression of the α subunit is evident in decidualised stromal cells, while its upregulation in the endometrium causes implantation failure, possibly through impaired endometrial receptivity and decidualization warranted by the inhibition of the activins. The expression of the βA and βB subunits is significantly upregulated in stromal cells during the secretory phase and is strongest in the late secretory phase when decidualization and embryo implantation take place [33]. Our results showed that α and βB subunits were decreased in the endometrium of RIF patients, while the degree of decline in βB subunit expression was greater than that of the α subunit, indicating that suppression of activin B may contribute to the pathology of RIF.

By transcriptome analyses, we found that the main downstream signalling pathways mediated by INHBB were immunomodulatory and inflammation-related pathways, including cytokine-cytokine receptor interactions, the TNF signalling pathway, the IL-17 signalling pathway, and the NF-κB signalling pathway. The involvement of the inflammation-related signalling pathway has been proven to be related to HESCs decidualization [35, 36]. INHBB was closely correlated with cancer-promoting signalling pathways, including the TGF-β signalling pathway, focal adhesion, breast cancer, and prostate cancer, which have been studied before [15]. Currently, the roles of INHBB in the cAMP signalling pathway, calcium signalling pathway, and phospholipase D signalling pathway are still unknown.

We then found that the downregulated expression of INHBB was consistent with ADCY1, which involved in regulating the cAMP signalling pathway. Numerous previous studies have documented that cAMP signalling pathways were important for decidualization [7, 37, 38]. The levels of intracellular cAMP were elevated during decidualization, and a sustained increase of intracellular cAMP is essential for HESCs decidualization [39]. cAMP is a common second messenger increased when extracellular ligands binding to G protein-coupled receptors. Following activation of membrane-bound adenylyl cyclase, ATP is converted to cAMP. Adenylate cyclase 1 (Adcy1) is the most upregulated gene in the oestrogen pathway during catagen [40]. While ADCY2 is another member of the adenylyl cyclase family. However, to date, no researchers have studied the biological function of ADCY in human endometrial decidualization. ADCY may be potential candidate genes for the identification of genetic variation influencing cow fertility [41, 42]. In a previous study, ADCY1 mRNAs were upregulated in the mid-secretory endometrium compared with proliferative endometria, suggesting that the above networks were closely related to endometrial receptivity [43]. Our results indicated that the knockdown of INHBB obviously decreased the expression of ADCY1 and the level of cAMP which caused impaired decidualization, while the addition of 8-Br-cAMP or forskolin rescued the level of PRL and IGFBP1. This suggested that INHBB regulates cAMP level by inducing the expression of ADCY1.

Our results also showed that reduced expression of INHBB caused a reduction in RYR2 (Supplementary Fig. 4 and 5), one of the components of a calcium channel that supplies calcium to cardiac muscle. A previous study revealed that the decrease in cytosolic Ca^2+^ levels increased cAMP concentrations, which intensified implantation-related gene expression and regulated the differentiation of endometrial stromal and glandular epithelial cells [44]. The relationship between INHBB and RYR2 in endometrial decidualization requires further exploration. Besides, cAMP levels depend not only on its production rate but also on its degradation rate by cyclic nucleotide phosphodiesterases [45]. Our results showed that INHBB knockdown is associated with the phospholipase D signalling pathway, indicating that there may be a complicated regulatory loop between INHBB and cAMP signalling pathway.

## Conclusions

In summary, our study showed significantly reduced expression of INHBB in endometrial stromal cells in women with RIF. INHBB knockdown in HESCs suppressed ADCY1-induced cAMP production and cAMP-mediated signalling, which caused impaired decidualization, indicating that INHBB is an important component of the decidualization process. We suggest that INHBB could serve not only as a potential prognostic biomarker for decidualization but also as a therapeutic application to increase the clinical pregnancy rate of patients with RIF.

## Supplementary Information

Below is the link to the electronic supplementary material.

Supplementary file1 (DOCX 2978 KB)

Supplementary file2 (RAR 49790 KB)

## Data Availability

The datasets presented in this study can be found in online repositories. The names of the repositories and accession numbers can be found in the article/supplementary material.
