# Supplementary material for: Downregulated INHBB in endometrial tissue of recurrent implantation failure patients impeded decidualization through the ADCY1/cAMP signalling pathway
Source: J Assist Reprod Genet. 2023 Mar 13;40(5):1135–46. doi: 10.1007/s10815-023-02762-7 (PMC10239411; doi:10.1007/s10815-023-02762-7)

**Supplementary information**

**Supplementary table 1**

|  | Control | RIF | *P* |
| --- | --- | --- | --- |
| n | 18 | 14 | - |
| Age (y) | 29.33 ± 2.20 | 30.64 ± 2.59 | 0.13 |
| BMI (kg/m^2^) | 22.58 ± 2.12 | 22.72 ± 2.54 | 0.87 |
| Number of embryo transfer | 1.78 ± 0.55 | 5.5 ± 2.02 | <0.0001 |

Clinical characteristics of women enrolled in the present study.

These data were expressed as mean ± SEM and the student’s t-test was used to compare the distribution of the data.

**Supplementary table 2**

| Gene Name | Sense primer | Antisense primers | NO. of fragment |
| --- | --- | --- | --- |
| INHBB | GAAATCATCAGCTTCGCCGAGAC | GGCAGGAGTTTCAGGTAAAGCC | 134bp |
| INHBA | GGCAAGTTGCTGGATTATAGTG | CTGAGAGTTGGGTACATCCTTT | 121bp |
| INHA | TCCCAAGCCATCCTTTTCCCAG | TCACCTGGCGGCTGCGTGTAT | 139bp |
| PRL | CACTACATCCATAACCTCTC | ATGCTGACTATCAGGCTCAG | 182bp |
| IGFBP1 | TATGATGGCTCGAAGGCTCTC | GTAGACGCACCAGCAGAGTC | 225bp |
| 18S | CGGCTACCACATCCAAGGAA | CTGGAATTACCGCGGCT | 186bp |

Primers used in this study.

**Supplementary Fig. 1** **Relative mRNA expression levels of inhibin/activin subunits.** **a** The relative mRNA expression of INHA was quantified using RT-qPCR. Expression levels are shown relative to 18S rRNA; CTL (n=14) and RIF (n=11). ***P*<0.01, using Student’s t test. **b** The relative mRNA expression of INHBA was quantified using RT-qPCR. Expression levels are shown relative to 18S rRNA; CTL (n=12) and RIF (n=6). NS: no statistical difference.


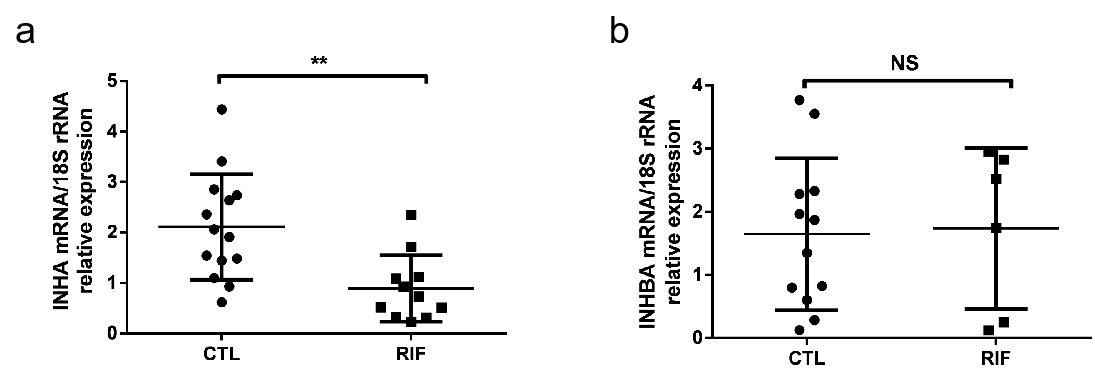


**Supplementary Fig. 2** INHBB protein expression in ICR mouse uteri during the peri-implantation period. Brightfield photomicrographs of representative uterine sections are shown. M, mesometrial pole; AM, antimesometrial pole. dpc, days post-coitum; dpc0.5 indicates the first day of identified vaginal plugs. Original image: scale bar=500 μm; upper and lower right magnification: scale bar=100 μm. Bar graph showing the mean IOD of the IHC images. Three samples were taken during each group and the last three randomly selected fields were scored and mean value was calculated as the sample value. ***P*<0.01.


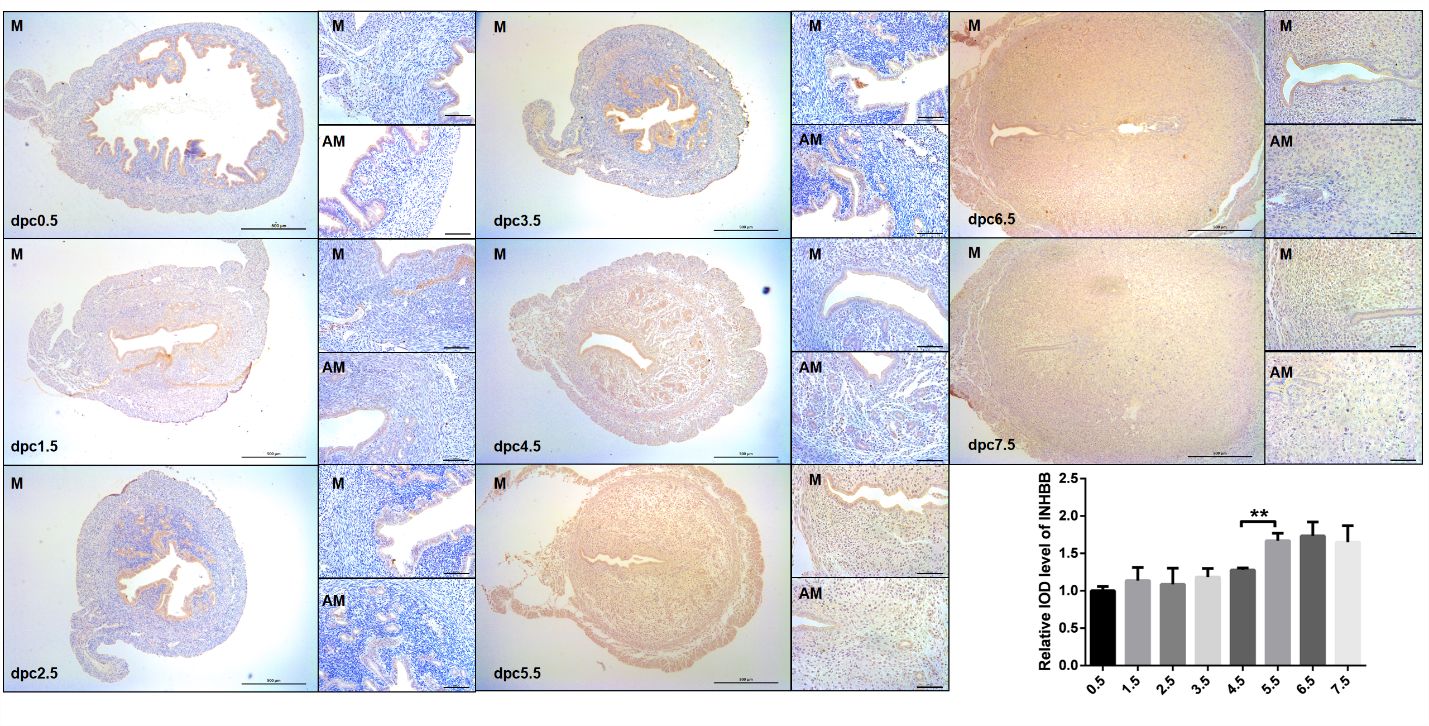


**Supplementary Fig. 3** Clustering of genes between different groups (data from Fig.5**c**). The gene numbers of different clusters are shown in the upper table.


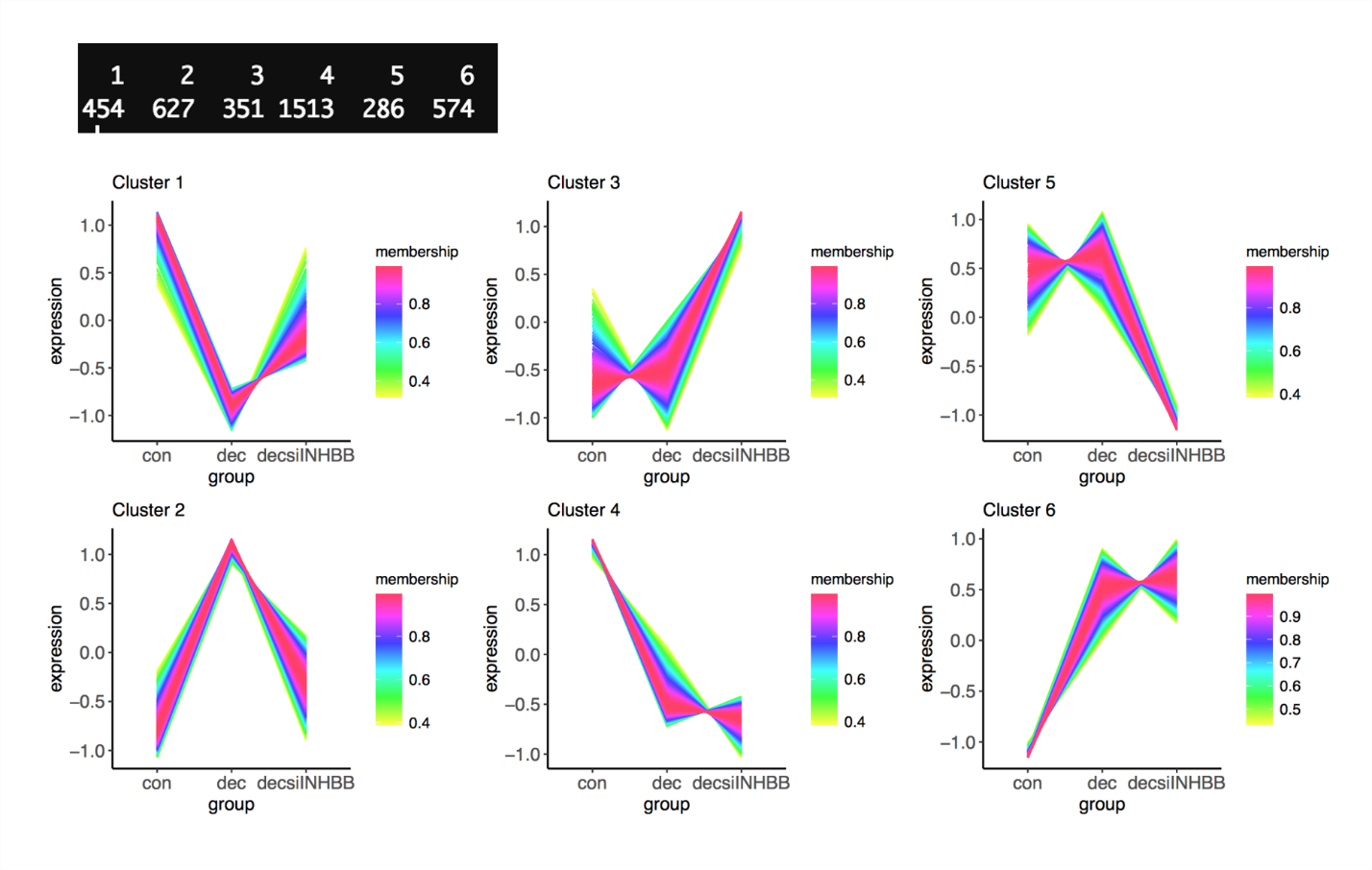


**Supplementary Fig. 4** Box plots of ADCY family (ADCY1-10) FRKM expression in HESCs treated with 0.5 mM 8Br-cAMP and 1 μM MPA for decidualization by RNA-seq among the control, decidualization (dec), si-INHBB+decidualization (decsiinhbb) groups.


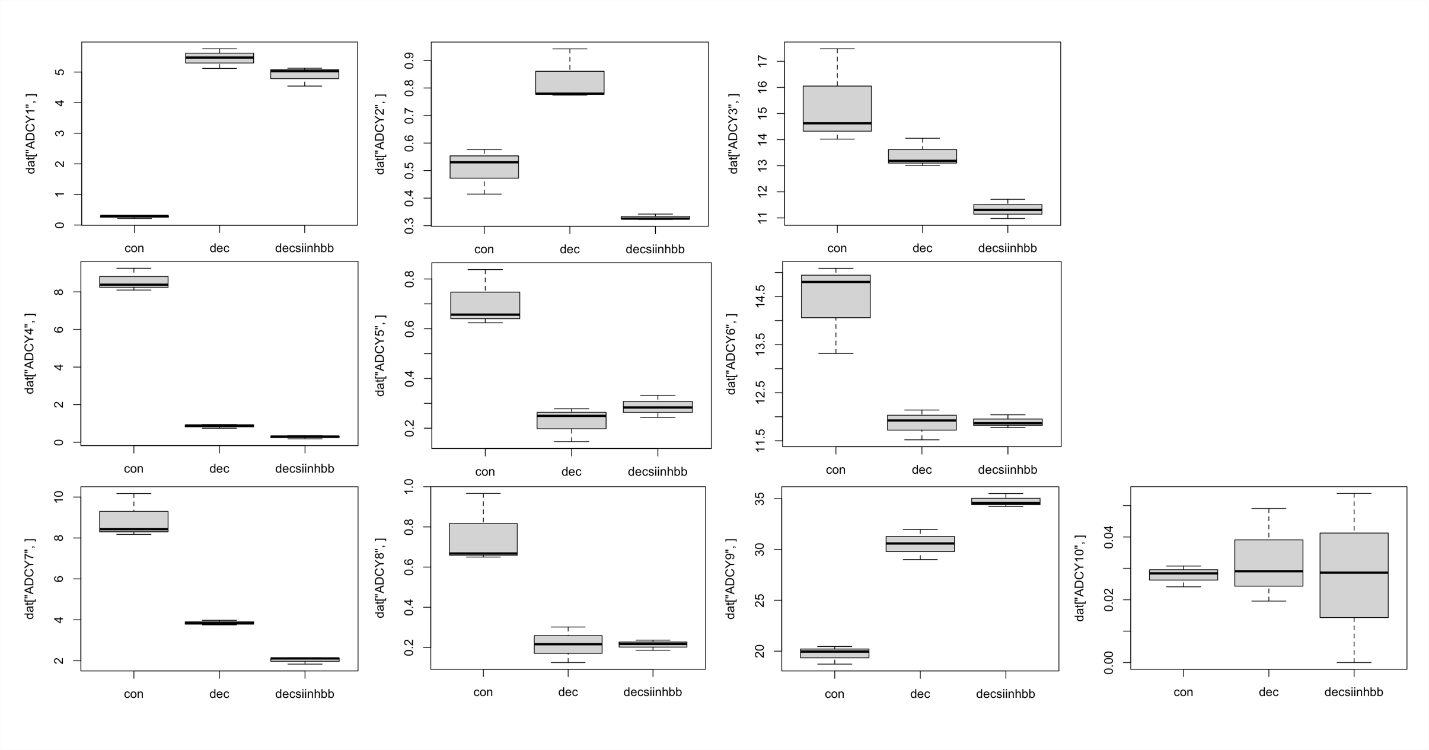


**Supplementary Fig. 5** Correlation analysis between INHBB mRNA expression levels and ADCY1/ADCY2/RYR2 concentration levels in RIF patients. **a-c** Pearson correlation analyse indicated that INHBB expression in the human endometrium (GSE111974) had a positive correlation with ADCY1 (**a**, *R^2^*=0.526, *P*<0.0001) and RYR2 (**c**, *R^2^*=0.526, *P*<0.0001) concentration levels. No correlations were found between INHBB mRNA expression and ADCY2 (**b**, *R^2^*=0.526, *P*<0.0001) concentrations. **d** Pearson correlation analysis of mid-secretory endometrial protein levels of INHBB and RYR2 mRNA levels (*R^2^*=0.526, *P*<0.0001) in all samples (n = 28).


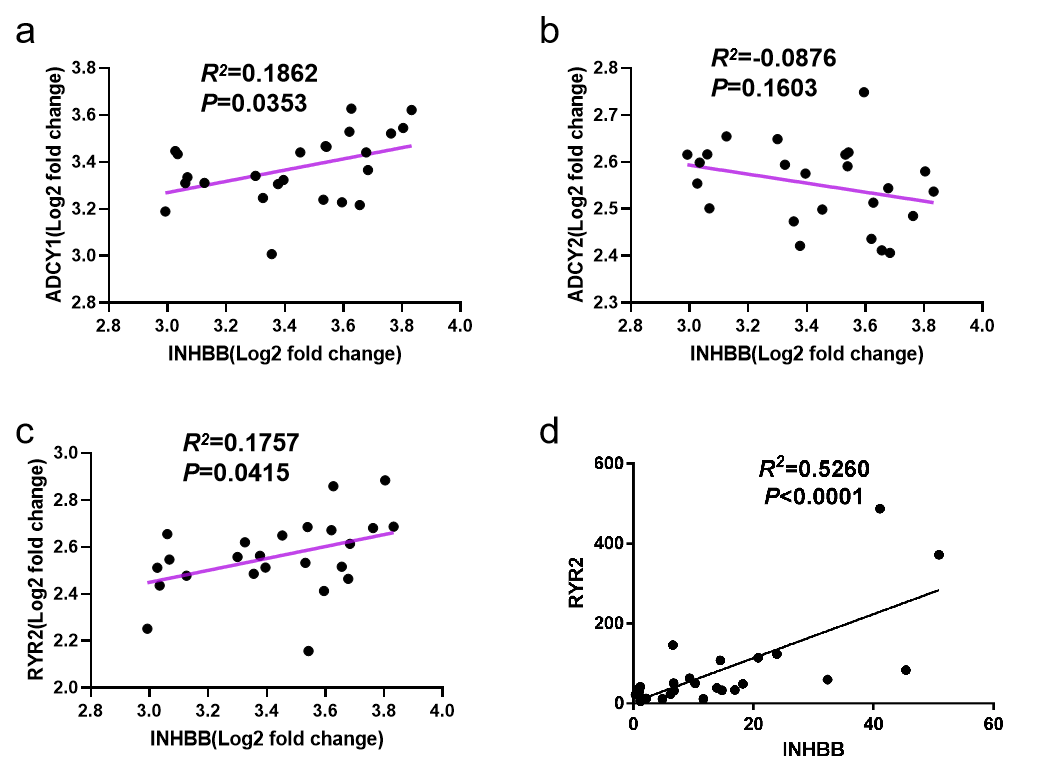


**Supplementary Fig. 6** Schematic representation of INHBB in the regulation of decidualization in RIF patients and fertile control groups.


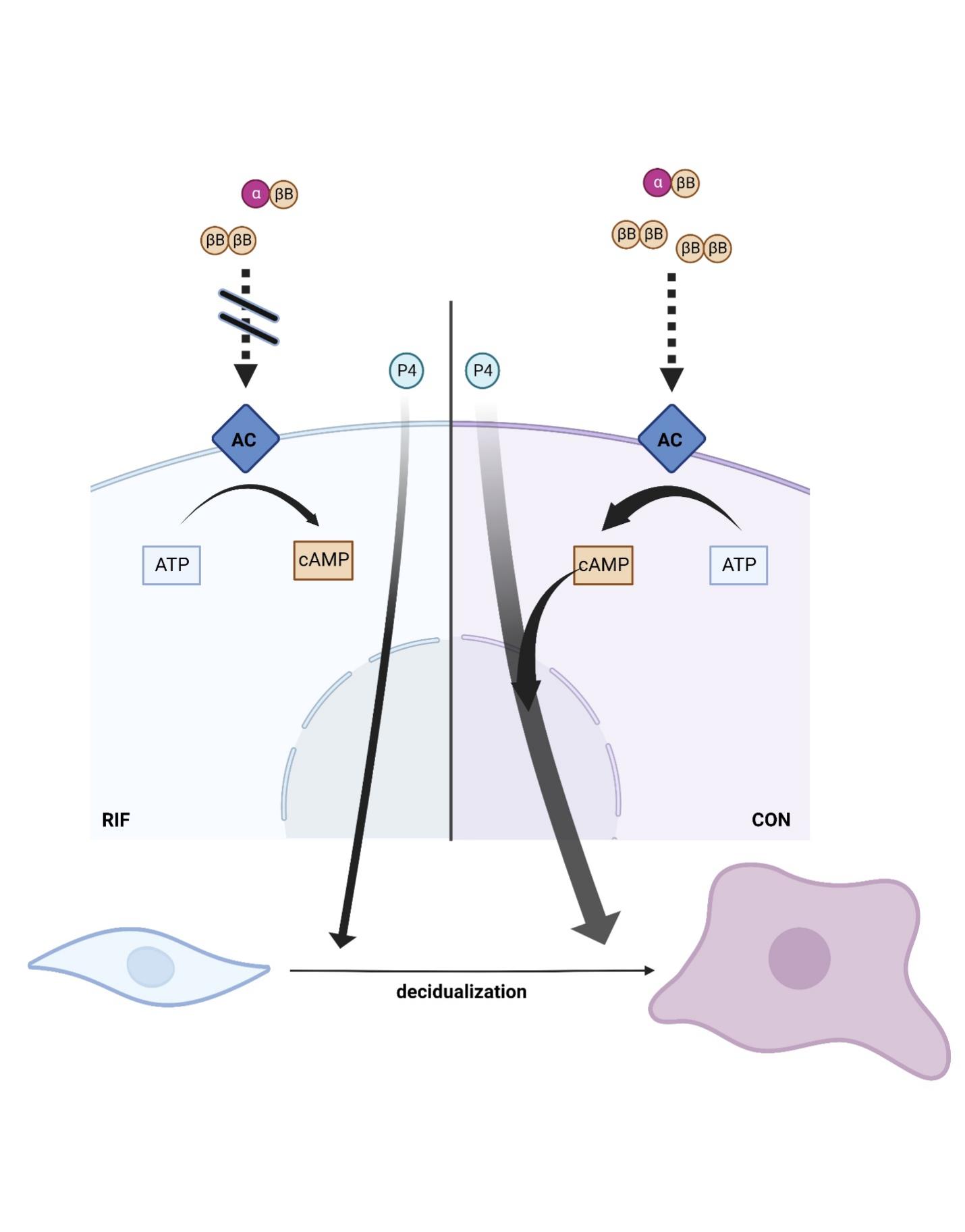

Supplement: Supplementary file 1 — Supplementary file1 (DOCX 2978 KB) [file 10815_2023_2762_MOESM1_ESM.docx]
